# Artificial insemination of boar semen doses prepared with a low-density colloid under field conditions

**DOI:** 10.3389/fvets.2025.1611751

**Published:** 2025-06-05

**Authors:** Athina Basioura, Ioannis A. Tsakmakidis, Jane M. Morrell, Theodoros Ntallaris

**Affiliations:** ^1^Farm Animals Clinic, School of Veterinary Medicine, Aristotle University of Thessaloniki, Thessaloniki, Greece; ^2^Clinical Sciences, Swedish University of Agricultural Sciences, Uppsala, Sweden

**Keywords:** antimicrobial resistance, artificial insemination, bacterial removal, boar, fertility outcomes, low-density colloid, reproduction, semen

## Abstract

**Introduction:**

Bacterial contamination of ejaculates during semen collection is practically inevitable, and antibiotics are a constituent of semen extenders. However, bacterial resistance to antibiotics is a serious problem. The present study investigated the effect of preparing semen by centrifugation using a low-density colloid during the preparation of semen doses under field conditions, as an alternative to the use of antibiotics.

**Methods:**

Five ejaculates from four boars were each divided into two aliquots: control semen doses, which were extended with a commercial Beltsville Thawing Solution (BTS) containing antibiotics (30 × 10^6^ spermatozoa/mL), and treated semen doses, which were processed with Porcicoll (300 × g; 20 min); the resulting sperm pellet was re-suspended in Beltsville Thawing Solution without antibiotics (30 × 10^6^ spermatozoa/mL). Sperm motility and kinematic variables were assessed for the control and treated semen doses using computer-assisted sperm analysis (CASA). Sows were divided into two groups and inseminated with either the control or treated semen doses. In the second round, after weaning the litters from the first round, the sows in the control group were inseminated with the treated semen doses and those in the treated group received the control semen doses. For all groups, the pregnancy rate, farrowing rate, litter size, number of live-born piglets, and number of weaned piglets were recorded.

**Results:**

No differences (*p* > 0.05) between the control and treated semen doses or between the rounds were observed for any CASA-assessed motility and kinematic variables. Pregnancy (*p* = 0.0271) and farrowing (*p* = 0.046) rates were higher in the sows in the control group compared to the treated group. No differences were observed in litter size, number of live-born piglets, and number of weaned piglets (*p* > 0.05) between the control and treated groups, and farrowing rates were the same for the sows in both rounds. Under the current experimental conditions, sperm quality was not impaired by treatment with Porcicoll. An interesting finding is that the sows were able to become pregnant again after insemination with semen doses lacking antibiotics, with no effect on reproductive output.

**Conclusion:**

In conclusion, artificial insemination (AI) with boar semen doses processed using Porcicoll, meeting modern requirements for alternatives to antibiotics, could be a promising state-of-the-art approach.

## 1 Introduction

Semen collection and processing in farm animals, including boars, are conducted under non-sterile conditions, making bacterial contamination of ejaculates practically inevitable. Bacteriospermia may compromise semen quality, resulting in reduced longevity of semen doses, sperm agglutination, impaired motility, toxicity from bacterial metabolic by-products, and other effects (1). Liquid-extended boar semen stored at 17°C for up to 10 days provides an ideal environment for bacterial proliferation (2). Antibiotics are a main constituent of semen extenders to prevent the growth of bacteria present in pig semen and, consequently, enhance reproductive performance (3). It is well documented that artificial insemination (AI) is the main technology used for farm animal breeding, including pigs, in almost all developed countries; therefore, a large volume of extenders supplemented with antibiotics is prepared and used. According to the WHO (4), “in several parts of the world, more than 50% in tonnage of all antimicrobial production is used in food-producing animals.” Therefore, any reduction in antibiotic usage must be considered beneficial.

Bacterial resistance to antibiotics is a serious problem for both human and animal health. The uncontrolled or intensive usage of antibiotics in previous decades is the main cause of this problem. Apart from the national or international regulations and recommendations for antibiotic use, including in semen extenders, there is a growing scientific effort to support innovation, research, and the development of new antimicrobial compounds and techniques. In this regard, semen centrifugation with colloids offers a promising perspective since it separates spermatozoa from bacteria through physical means. It has been examined for bacteria removal as a potential alternative to the conventional antibiotics included in semen extenders (5). In this context, single-layer centrifugation with a low- or high-density colloid has been successfully used for bacterial removal from boar semen (6), stallion semen (5), pony stallion semen (7), and bull (8) semen, with no detrimental effect on sperm quality. Indeed, boar sperm chromatin stability was favored by low-density colloid centrifugation, and sperm quality under long-term storage conditions at 17° C was enhanced (9, 10).

In addition to the above-mentioned experimental protocols, where small volumes of semen were processed, its effectiveness has also been proven for processing large volumes of boar or stallion semen under *in vitro* conditions (11). From a practical perspective, the latter results are interesting since large volumes of boar semen are needed for the production of AI semen doses, and any method designed to replace antibiotics must be capable of processing large volumes of ejaculate efficiently. However, there is limited information regarding the effect of processing a whole ejaculate using this technique to produce standard insemination doses on pig farm reproductive performance.

The aim of the present study was to investigate the effect of preparing AI boar semen doses using a single-layer centrifugation protocol with a low-density colloid (LSLC) on *in vivo* fertility outcomes, including pregnancy rate, farrowing rate, litter size, and the number of live-born and weaned piglets, compared to routine processing using extenders containing antibiotics. The study also aimed to examine the effect of insemination without antibiotics on the future reproductive efficiency of sows. The LSLC technique has previously been successfully used for bacterial removal (6) and has been scaled up under laboratory conditions. Therefore, this study serves as a follow-up to examine its effectiveness under field conditions.

## 2 Materials and methods

**Figure 1:**
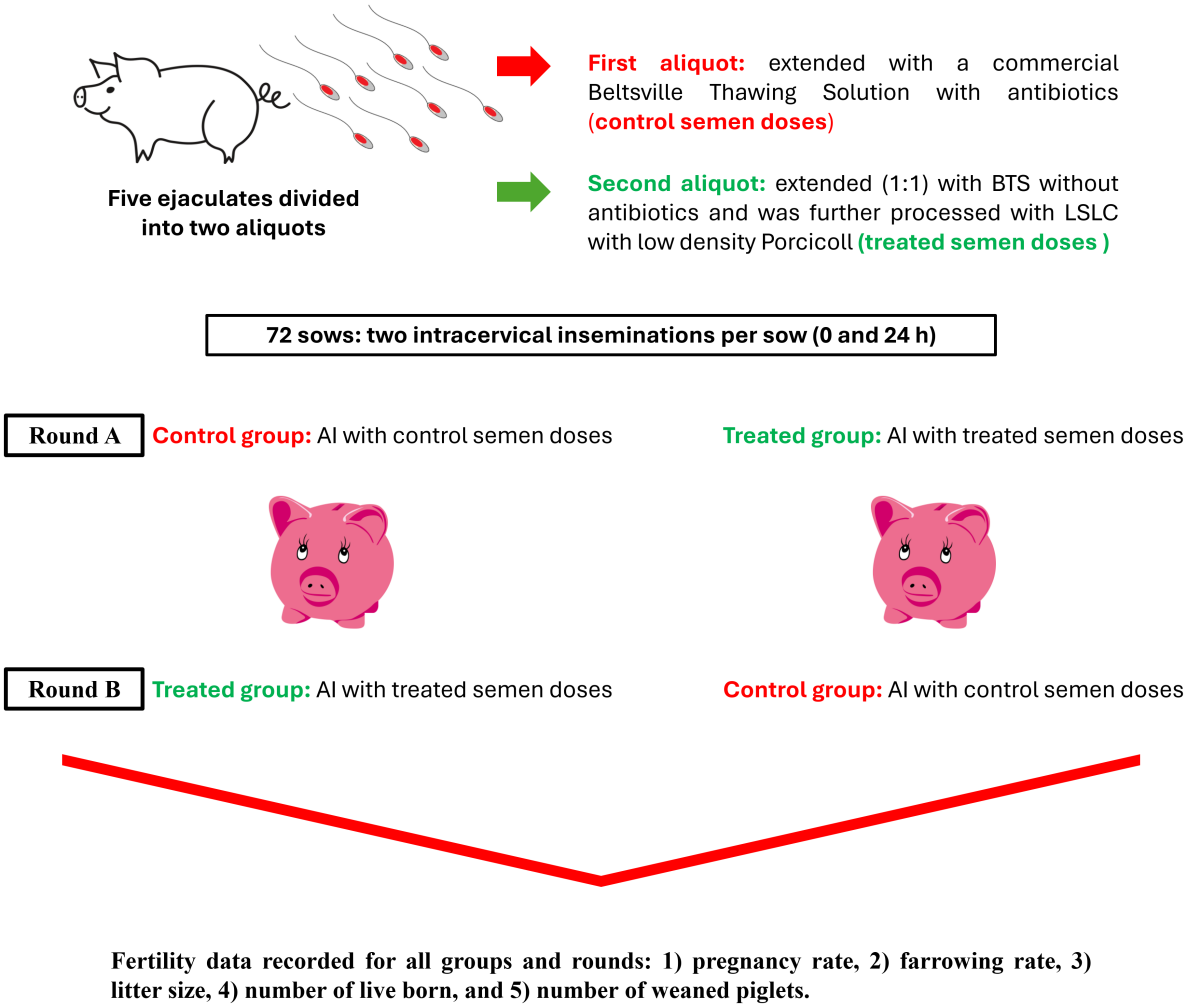
Experimental design. Boar ejaculates were split, with one part extended in Beltsville Thawing Solution with antibiotics (Controls) and the other part extended without antibiotics before being prepared by centrifugation through a single layer of a low-density colloid (Porcicoll). The sperm pellet was then resuspended in extenders without antibiotics. Both sets of the sperm samples were prepared as insemination doses and used to inseminate 72 sows on the same farm. BTS, Beltsville Thawing Solution; LSLC, Single-Layer Centrifugation using a low-density colloid; AI, artificial insemination.

This study was conducted on an industrial 700-sow capacity pig farm in Northern Greece. Since no animal manipulation was involved and owner consent was obtained, ethical approval was not necessary. All pigs were housed and handled according to national and international regulations for farm animals. The experimental design is shown in Figure 1.

### 2.1 Animals

A total of four boars and 72 sows were included in the study. All animals were housed under controlled environmental conditions. They were under veterinary supervision throughout the study period, properly vaccinated, and dewormed according to the farm’s routine disease-preventive animal health care program. Their nutrition followed the standards set by the Nutrient Requirements of Swine, and they had ad libitum access to water. All boars (Pietrain, Duroc×Pietrain; 1.5–2.5 years of age) were sexually mature, housed in individual pens, routinely used for semen dose production on the farm, and had approved fertility. The sows (Large White, Landrace, Large White×Landrace; Parity≥2) were of proven fertility and used for routine insemination on the farm. Estrus detection was performed once per day, every 24 h; a boar was used to assess the standing reflex.

### 2.2 Semen collection and processing

A total of five ejaculates (one or two per boar) were collected from the four boars active in the routine AI program of the farm, using the gloved-hand method by a technician at the farm. All semen samples meeting the minimum quality criteria for AI semen dose production [semen volume: > 200 mL; sperm concentration: > 200 × 10^6^ spermatozoa/mL; total motility: > 70%; viability: > 75%; normal morphology: > 80%, (12)] were further processed. Specifically, each ejaculate was divided into two aliquots. The first one was extended with a commercial Beltsville Thawing Solution (BTS) containing antibiotics (Minitube^®^, Germany), and AI semen doses of 100 mL were prepared with a sperm concentration of 30 × 10^6^ spermatozoa/mL (control semen doses). The second aliquot was extended (1:1) with BTS without antibiotics (Minitube^®^, Germany) and was further processed using a single-layer centrifugation protocol (LSLC) with low-density Porcicoll. Specifically, 80 mL of the semen extended in BTS without antibiotics was centrifuged on 60 mL of Porcicoll (300 × g; 20 min), and finally, the sperm pellet was re-suspended in BTS without antibiotics (volume of 100 mL; 30 × 10^6^ spermatozoa/mL; treated semen doses). The control and treated semen doses were used for insemination according to the experimental design described below, and a volume of 10 mL from each sample was transferred at 17° C to the laboratory, where motility and kinematic variables were assessed using computer-assisted sperm analysis (CASA) for both groups.

### 2.3 Assessment of CASA-assessed sperm motility and kinematic variables

Sperm motility and kinematic variables were assessed using CASA (CASA; Sperm Class Analyzer^®^, Microptic S. L., Automatic Diagnostic Systems, Spain). A microscope (100×; AXIO Scope A1, Zeiss, Germany) with a heating stage (at 37° C) and a preheated (37° C) Makler chamber (Makler^®^ counting chamber, 10 μm deep, Sefi Medical Instruments, Israel) were used for the CASA. Aliquots of 10 μL of the semen sample were placed on the Makler chamber, and duplicate assessments were performed for both groups using the following settings.

The percentage of total motility, progressive and non-progressive spermatozoa, and rapid/medium/slow and static movement spermatozoa was determined (static < 10 < slow 25 < medium < 45 μm/s; %). The following CASA kinematic parameters were evaluated: curvilinear velocity-VCL (μm/s), straight line velocity-VSL (μm/s), average path velocity-VAP (μm/s), amplitude of lateral head displacement-ALH (μm), beat/cross-frequency-BCF (Hz), linearity-LIN (VSL/VCL × 100), straightness-STR (VSL/VAP × 100), wobble-WOB (VAP/VCL × 100), and hyperactivation (VSL > 97 μm/s, ALH > 3.5 μm, LIN < 0.32; %). The analysis was conducted using the Sperm Class Analyzer software (SCA^®^ v.6.1., Microptic S. L., Spain) with the following configuration settings: at least 8 fields or >500 spermatozoa were assessed per sample, frame rate was 25 frames/s, the region of particle control was defined at 10–18 μm, progressive movement was >45% of the parameter STR, circumferential movement was <50% of the parameter LIN, the depth of field was 10 μm, and the temperature of the microscope plate was set to 37° C.

### 2.4 Experimental design of the study

Round A: The sows included in round A were randomly selected based on their estrus detection on the first day of the experiment. In total, 50 sows were included and divided into two groups (25 sows per group). For both groups, two intracervical insemination procedures were conducted per sow (AI at 0 and 24 h). The first group was inseminated with the control semen doses (control group), and the second group with the treated semen doses (treated group). The control and treated semen doses were prepared on the first day of the experiment; some were used for AI at 0 h, and the remaining doses were stored at 17° C for AI at 24 h.

Round B: After weaning the litters from the first round, the sows were examined for estrus onset for the second round of insemination, using a cross-over design. This time, the first group of sows received the treated sperm doses, and the second group received the control doses. The majority of the sows from round A were included in round B, except for 22 sows that were replaced for unexpected reasons (loss of the sow or no estrus detection on the day of the experiment).

In both rounds, the sows were scanned using ultrasonography for pregnancy diagnosis 28 days post-mating. The piglets were weaned 28 days after parturition, and the sows were examined for estrus onset over the following 7 days. According to the reproductive management protocol of the farm, no hormonal treatment for estrus synchronization was administered. The following fertility data were recorded for all groups: (1) pregnancy rate, (2) farrowing rate, (3) litter size, (4) the number of live-born piglets, and (5) the number of weaned piglets. When the experiments were completed, the involved sows were included in the farm’s routine AI program. The same fertility data for the first post-experimental AI (pregnancy or return to estrus) in the sows were recorded to examine any possible side effects on the sow’s subsequent reproductive performance.

### 2.5 Statistical analysis

Statistical analyses were conducted using the SAS® software (version 9.4; SAS Institute Inc., Cary, NC). The data were tested for normal distribution using the Kolmogorov–Smirnov test. Descriptive statistics (mean, standard deviation, min, max) were calculated using the MEANS and SGPLOT procedures in the software. The data on boar sperm properties were analyzed using PROC MIXED in the SAS software. The data that deviated from a normal distribution were log-transformed (VAP, non-progressive motility). However, to improve clarity, avoid redundancy, and facilitate interpretation, the respective log-transformed values are referred to and presented as untransformed values throughout the article.

Least-squares means (LSM ± SEM) estimated by the models were adjusted using the Scheffé method for multiple post-ANOVA comparisons. The fixed effects included Round (*n* = 2), Treatment Group (*n* = 2), Boar (*n* = 4), and the interaction Group*Boar.

The alpha value for this experiment was set at 5%, and *p*-values were evaluated based on the selected alpha value. Differences with 0.05 < *p* ≤ 0.10 were considered indicative of a trend.

## 3 Results

### 3.1 Sperm motility and kinematics evaluated using CASA

There were no differences (*p* > 0.05) between the control and treated semen doses (Table 1) or rounds A and B (Table 2) for any of the sperm motility and kinematic variables assessed using CASA. An effect of boar on the CASA-assessed variables was observed (Table 3). The percentage of non-progressive spermatozoa was lower (*p* = 0.0123) for boar 3 compared to boar 1, while VSL was higher (*p* = 0.0194) for boar 3 compared to boar 1 (Table 3). For VCL (*p* = 0.0028), VAP (*p* = 0.0052), ALH (*p* = 0.0025), and hyperactivation (*p* = 0.0095), higher values were obtained for boar 3 compared to boars 1 and 2 (Table 3). A difference was observed for STR (*p* = 0.0153), with boar 2 having higher values compared to boar 4 (Table 3). Table 4 shows the sperm motility and kinematic variables assessed using CASA for the control and treated semen doses per round.

**Table 1:** Sperm motility and kinematic variables evaluated using CASA in the control and treated semen dose groups.

CASA-assessed variables	Control	Treated	*p*-value
Totmot (%)	92.12 ± 3.96	94.64 ± 1.21	>0.05
Static (%)	7.88 ± 3.96	5.36 ± 1.21	
Nonpro (%)	28.58 ± 5.09	30.32 ± 4.43	
Pro (%)	63.54 ± 7.71	64.32 ± 5.26	
Rapid (%)	42.00 ± 11.17	42.20 ± 5.87	
Medium (%)	26.40 ± 3.97	27.03 ± 2.54	
Slow (%)	23.72 ± 5.54	25.40 ± 3.60	
VCL (μm/s)	56.16 ± 13.02	55.77 ± 9.83	
VSL (μm/s)	27.13 ± 5.27	27.34 ± 3.57	
VAP (μm/s)	42.11 ± 11.80	41.10 ± 8.10	
LIN (%)	46.71 ± 3.03	46.20 ± 3.07	
STR (%)	66.04 ± 4.03	63.72 ± 3.71	
WOB (%)	68.41 ± 3.93	68.29 ± 1.77	
ALH (μm)	2.18 ± 0.32	2.25 ± 0.30	
BCF (Hz)	6.19 ± 0.15	5.73 ± 0.21	
Hyper (%)	1.68 ± 1.12	1.91 ± 1.06	

Totmot: total motility (%), Nonpro: non-progressive spermatozoa (%), Pro: progressive spermatozoa (%), Rapid-Medium-Slow: rapid, medium, and slow spermatozoa (%; 10 < slow<25 < medium<45 < rapid μm/s), VCL: curvilinear velocity (μm/s), VSL: straight line velocity (μm/s), VAP: average path velocity (μm/s), LIN: linearity (VSL/VCL x 100), STR: straightness (VSL/VAP x 100), WOB: wobble (VAP/VCL x 100), ALH: amplitude of lateral head displacement (μm), BCF: beat/cross-frequency (Hz), and Hyper: hyperactive (%).

**Table 2:** Sperm motility and kinematic variables evaluated using CASA in rounds A and B.

CASA-assessed variables	Round A	Round B	*p*-value
Totmot (%)	90.92 ± 4.67	95.02 ± 1.37	>0.05
Static (%)	9.08 ± 4.67	4.98 ± 1.37	
Nonpro (%)	24.02 ± 6.56	33.07 ± 2.62	
Pro (%)	66.91 ± 10.10	61.95 ± 3.93	
Rapid (%)	46.60 ± 12.75	39.11 ± 6.05	
Medium (%)	23.45 ± 3.89	28.90 ± 2.53	
Slow (%)	20.88 ± 6.16	27.01 ± 3.33	
VCL (μm/s)	67.85 ± 17.06	48.05 ± 5.44	
VSL (μm/s)	32.83 ± 6.31	23.51 ± 2.02	
VAP (μm/s)	52.82 ± 14.45	34.13 ± 5.03	
LIN (%)	47.57 ± 3.66	45.71 ± 2.60	
STR (%)	63.81 ± 4.81	65.60 ± 3.31	
WOB (%)	72.01 ± 2.42	65.91 ± 2.66	
ALH (μm)	2.51 ± 0.46	2.02 ± 0.16	
BCF (Hz)	5.89 ± 0.22	6.01 ± 0.20	
Hyper (%)	3.21 ± 1.58	0.85 ± 0.39	

Totmot: total motility (%), Nonpro: non-progressive spermatozoa (%), Pro: progressive spermatozoa (%), Rapid-Medium-Slow: rapid, medium, and slow spermatozoa (%; 10 < slow<25 < medium<45 < rapid μm/s), VCL: curvilinear velocity (μm/s), VSL: straight line velocity (μm/s), VAP: average path velocity (μm/s), LIN: linearity (VSL/VCL x 100), STR: straightness (VSL/VAP x 100), WOB: wobble (VAP/VCL x 100), ALH: amplitude of lateral head displacement (μm), BCF: beat/cross-frequency (Hz), and Hyper: hyperactive (%).

**Table 3:** Effect of boar on the CASA-assessed sperm motility and kinematic variables.

CASA-assessed variables	Boar 1	Boar 2	Boar 3	Boar 4	*p*-value
Totmot (%)	89.34 ± 4.15	93.15 ± 2.45	96.70 ± 0.40	98.37 ± 0.95	>0.05
Static (%)	10.66 ± 4.15	6.86 ± 2.46	3.30 ± 0.40	1.64 ± 0.95	>0.05
Nonpro (%)	**36.03 ± 2.53** ^**a**^	**34.87 ± 3.09** ^**a, b**^	**12.79 ± 0.80** ^**b**^	**27.53 ± 3.69** ^**a, b**^	**0.0123**
Pro (%)	53.31 ± 4.37	58.28 ± 5.54	83.92 ± 1.20	70.84 ± 4.63	>0.05
Rapid (%)	29.16 ± 4.71	28.90 ± 2.89	66.46 ± 11.42	56.85 ± 0.79	>0.05
Medium (%)	28.93 ± 1.23	33.72 ± 2.22	19.77 ± 7.91	22.25 ± 4.36	>0.05
Slow (%)	31.26 ± 3.00	30.52 ± 2.65	10.49 ± 3.11	19.27 ± 4.20	>0.05
VCL (μm/s)	**40.10 ± 2.39** ^**a**^	**38.11 ± 2.14** ^**a**^	**96.90 ± 7.02** ^**b**^	**64.62 ± 0.52** ^**a, b**^	**0.0028**
VSL (μm/s)	**21.53 ± 1.81** ^**a**^	**21.07 ± 1.20** ^**a, b**^	**43.63 ± 2.44** ^**b**^	**28.43 ± 2.17** ^**a, b**^	**0.0194**
VAP (μm/s)	**27.53 ± 2.09** ^**a**^	**26.22 ± 0.43** ^**a**^	**77.27 ± 7.49** ^**b**^	**49.49 ± 1.24** ^**a, b**^	**0.0046**
LIN (%)	48.46 ± 3.59	51.36 ± 4.97	43.08 ± 0.04	40.93 ± 3.18	>0.05
STR (%)	**70.10 ± 2.20** ^**a, b**^	**72.34 ± 1.22** ^**a**^	**56.32 ± 0.93** ^**a, b**^	**55.56 ± 2.31** ^**b**^	**0.0153**
WOB (%)	64.56 ± 3.46	66.30 ± 4.46	74.83 ± 1.75	71.52 ± 2.53	>0.05
ALH (μm)	**1.79 ± 0.08** ^**a**^	**1.73 ± 0.14** ^**a**^	**3.29 ± 0.04** ^**b**^	**2.48 ± 0.12** ^**a, b**^	**0.0025**
BCF (Hz)	5.91 ± 0.22	5.93 ± 0.61	6.18 ± 0.34	5.88 ± 0.30	>0.05
Hyper (%)	**0.75 ± 0.39** ^**a**^	**−0.25 ± 0.68** ^**a**^	**6.16 ± 0.68** ^**b**^	**1.3 ± 0.68** ^**a, b**^	**0.0095**

Totmot: total motility (%), Nonpro: non-progressive spermatozoa (%), Pro: progressive spermatozoa (%), Rapid-Medium-Slow: rapid, medium, and slow spermatozoa (%; 10 < slow<25 < medium<45 < rapid μm/s), VCL: curvilinear velocity (μm/s), VSL: straight line velocity (μm/s), VAP: average path velocity (μm/s), LIN: linearity (VSL/VCL x 100), STR: straightness (VSL/VAP x 100), WOB: wobble (VAP/VCL x 100), ALH: amplitude of lateral head displacement (μm), BCF: beat/cross-frequency (Hz), and Hyper: hyperactive (%).

The values in bold are statistically significant. ^a,b^ refer to values that are statistically different.

**Table 4:** CASA-assessed sperm motility and kinematic variables for the control and treated semen doses in rounds A and B.

CASA-assessed variables	Control		Treated		*p*-value
	Round A	Round B	Round A	Round B	
Totmot (%)	87.13 ± 9.97	95.45 ± 2.28	94.71 ± 1.59	94.59 ± 2.01	>0.05
Static (%)	12.87 ± 9.97	4.55 ± 2.28	5.29 ± 1.59	5.41 ± 2.01	
Nonpro (%)	22.43 ± 10.44	32.68 ± 5.39	25.61 ± 12.02	33.45 ± 2.25	
Pro (%)	64.70 ± 20.41	62.76 ± 7.66	69.11 ± 13.61	61.13 ± 4.23	
Rapid (%)	48.55 ± 29.33	37.64 ± 10.28	44.65 ± 10.40	40.57 ± 8.69	
Medium (%)	20.00 ± 8.14	30.67 ± 2.76	26.90 ± 0.77	27.12 ± 4.62	
Slow (%)	18.60 ± 11.22	27.13 ± 6.76	23.17 ± 9.58	26.89 ± 3.14	
VCL (μm/s)	69.87 ± 34.06	47.02 ± 8.60	65.83 ± 24.05	49.07 ± 8.55	
VSL (μm/s)	34.25 ± 11.81	22.38 ± 4.21	31.41 ± 9.79	24.63 ± 1.21	
VAP (μm/s)	56.12 ± 28.64	32.77 ± 9.05	49.53 ± 20.26	35.49 ± 6.54	
LIN (%)	50.76 ± 7.64	44.01 ± 1.40	44.38 ± 1.34	47.42 ± 5.38	
STR (%)	65.89 ± 10.51	66.14 ± 4.17	61.73 ± 4.48	65.06 ± 6.09	
WOB (%)	74.90 ± 1.68	64.09 ± 5.22	69.12 ± 3.96	67.73 ± 2.20	
ALH (μm)	2.45 ± 0.88	2.00 ± 0.18	2.56 ± 0.69	2.04 ± 0.30	
BCF (Hz)	6.13 ± 0.38	6.23 ± 0.17	5.65 ± 0.19	5.79 ± 0.36	
Hyper (%)	3.52 ± 2.52	0.46 ± 0.46	2.90 ± 2.90	1.25 ± 0.63	

Totmot: total motility (%), Nonpro: non-progressive spermatozoa (%), Pro: progressive spermatozoa (%), Rapid-Medium-Slow: rapid, medium, and slow spermatozoa (%; 10 < slow<25 < medium<45 < rapid μm/s), VCL: curvilinear velocity (μm/s), VSL: straight line velocity (μm/s), VAP: average path velocity (μm/s), LIN: linearity (VSL/VCL x 100), STR: straightness (VSL/VAP x 100), WOB: wobble (VAP/VCL x 100), ALH: amplitude of lateral head displacement (μm), BCF: beat/cross-frequency (Hz), and Hyper: hyperactive (%).

### 3.2 Fertility data

**Figure 2:**
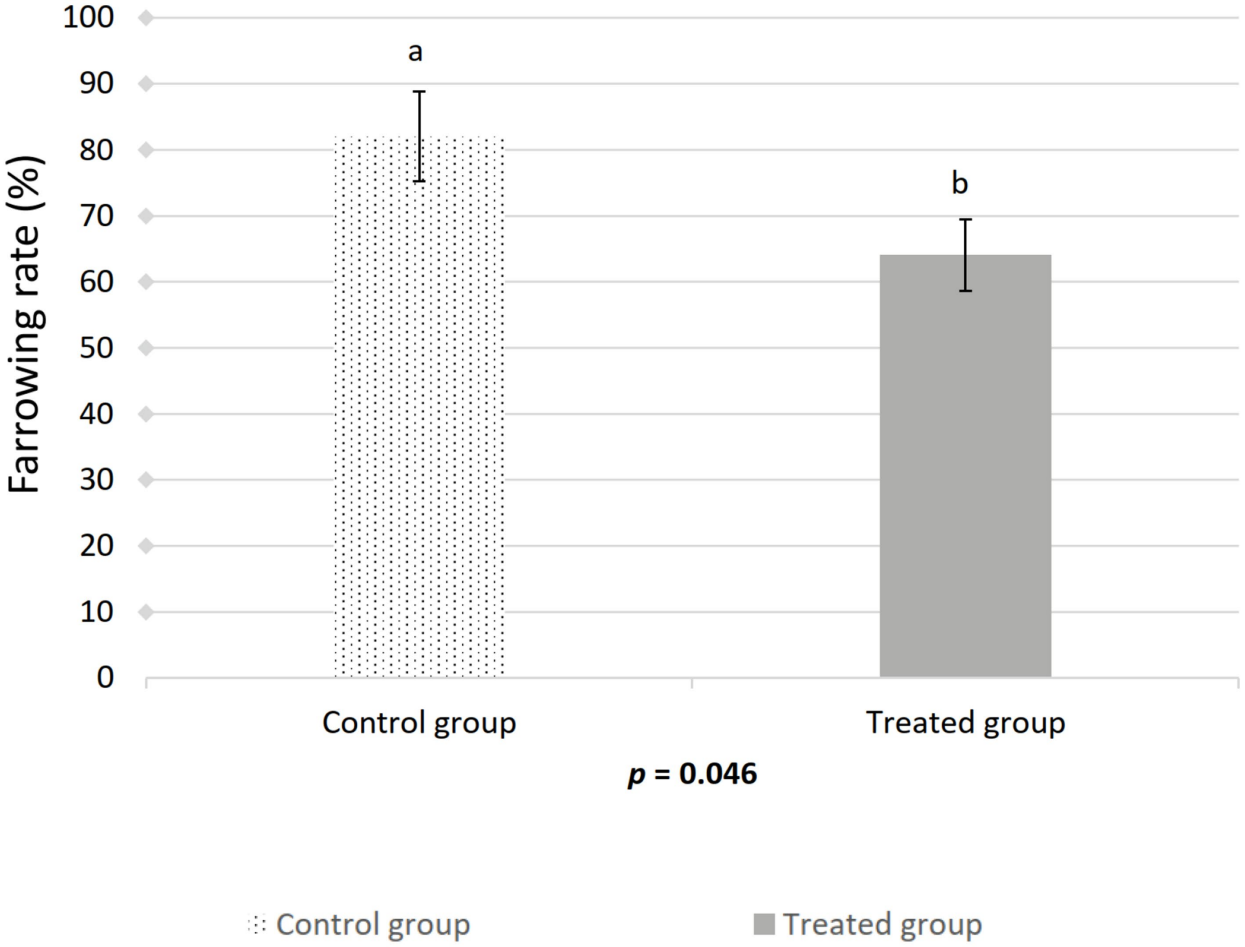
Effect of artificial insemination boar semen dose preparation using a single-layer centrifugation protocol with a low-density colloid (Porcicoll) on the farrowing rate. Control group: sows inseminated with the control semen doses; Treated group: sows inseminated with the treated semen doses. Values are expressed as LSM ± SEM. Different subscripts (a, b) indicate differences between the groups (*p* < 0.05).

**Figure 3:**
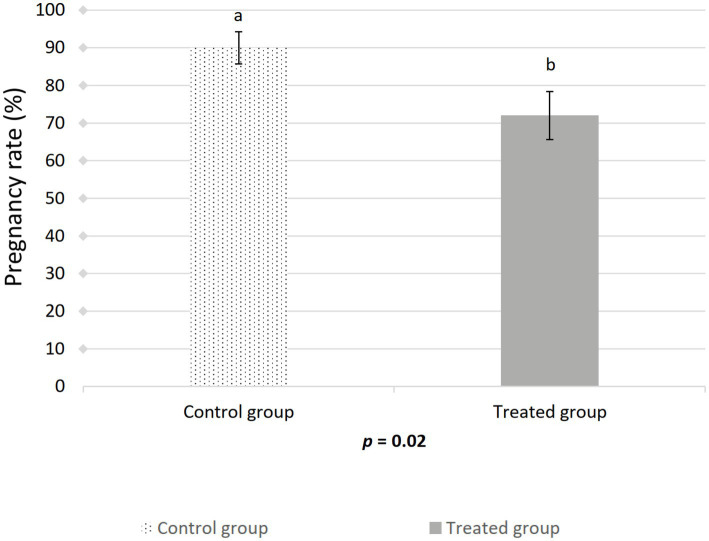
Effect of artificial insemination boar semen dose preparation using a single-layer centrifugation protocol with a low-density colloid (Porcicoll) on the pregnancy rate. Control group: sows inseminated with the control semen doses; Treated group: sows inseminated with the treated semen doses. Values are expressed as LSM ± SEM. Different subscripts (a, b) indicate differences between the groups (*p* < 0.05).

**Figure 4:**
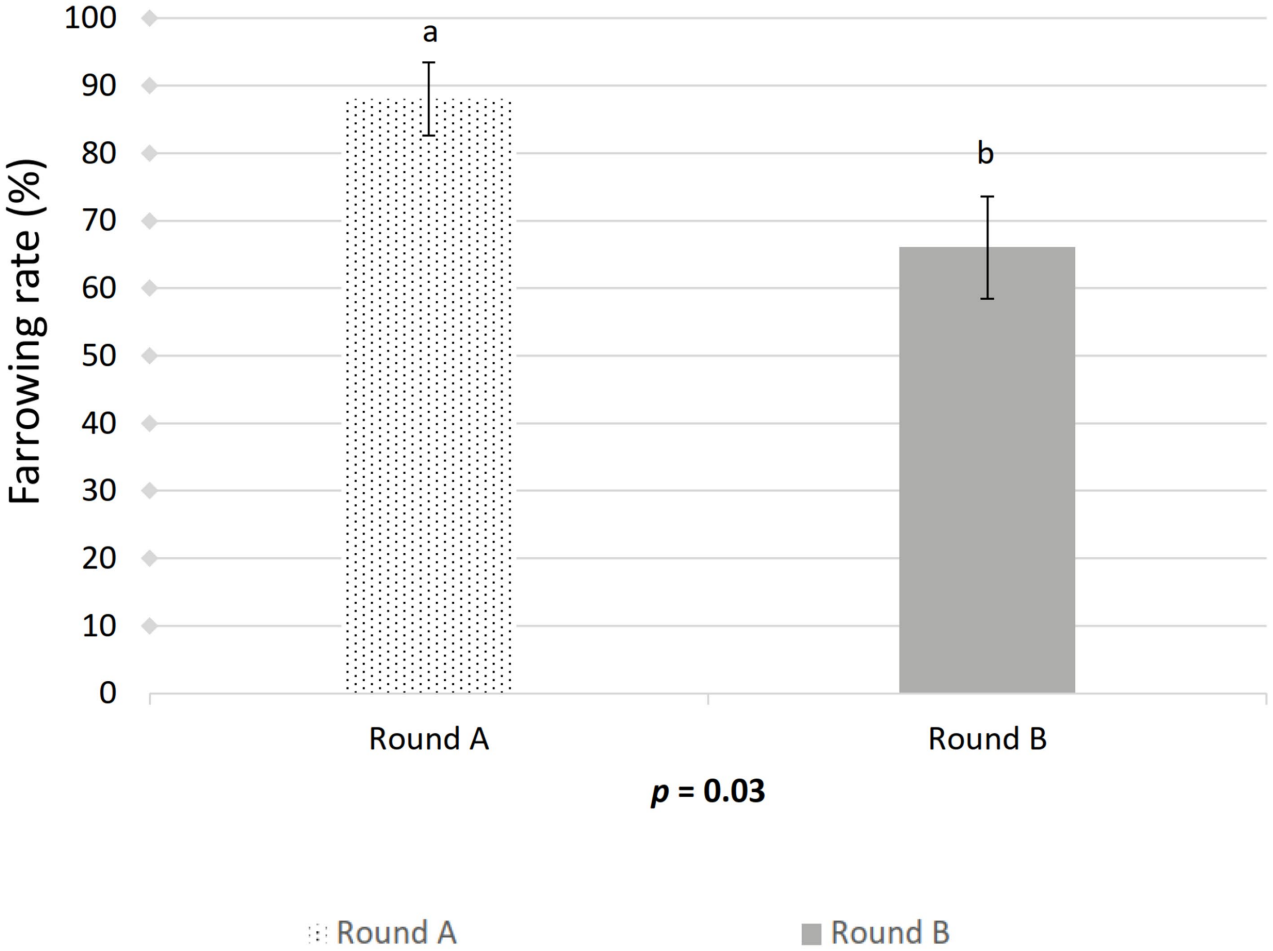
Effect of artificial insemination boar semen dose preparation using a single-layer centrifugation protocol with a low-density colloid (Porcicoll) on the farrowing rate. Sows were inseminated with the control or treated semen doses (Round A) or vice versa (Round B). Values are expressed as LSM ± SEM. Different subscripts (a, b) indicate differences between the groups (*p* < 0.05).

The farrowing (*p* = 0.046) and pregnancy (*p* = 0.0271) rates were higher in the sows included in the control group compared to the treated group (Figures 2, 3). There was no effect of boar on the pregnancy or farrowing rate, but an effect of round was noticed only for the farrowing rate (Figure 4), which was higher (*p* = 0.03) in round A (88.04 ± 5.4%) compared to round B (66.02 ± 7.6%). Based on the analysis of the data from the sows included in the crossover experiment (data not shown), the sows exhibited the same farrowing rate regardless of the round, but there was a difference between the boars.

Although the number of live-born piglets was higher (*p* = 0.04) in round A than in round B (Table 5), no differences were observed in litter size or the number of live-born and weaned piglets (*p* > 0.05) between the control and treated sow groups (Table 6). The analysis of interactions between the boars and sows revealed no statistical differences (*p* > 0.05) for any of the examined fertility variables (Table 7). From a biological point of view, only three of 10 sows became pregnant when one boar’s treated semen doses were used in the first round. However, the same three sows became pregnant with the control doses in the next round as well. Although this effect disappeared when considering the overall pregnancy rate between the control and treated semen, this finding could suggest a potential biological interaction between that boar’s semen and the sows. However, a large number of events is necessary to support this argument statistically. No effect of boar on the number of live-born and weaned piglets was observed (*p* > 0.05; Table 8), although there was a tendency for differences in litter size, and the lowest values were recorded for boar 3 (*p* = 0.07; Table 8).

**Table 5:** Litter size and the number of live-born and weaned piglets in experiments A and B.

Round	Litter size	Live-born	Weaned
A	14.87 ± 1.02	14.77 ± 1.03^a^	12.99 ± 0.97
B	12.06 ± 0.94	11.70 ± 0.95^b^	11.20 ± 0.90

Different subscripts (a, b) for the number of live-born piglets indicate differences between the rounds (*p* = 0.04). No differences were observed in litter size and the number of weaned piglets (*p* > 0.05).

**Table 6:** Litter size and the number of live-born and weaned piglets in the control and treated sow groups.

Sows	Litter size	Live-born	Weaned	*p*-value
Control group	13.56 ± 0.62	13.32 ± 0.63	12.24 ± 0.59	group > 0.05
Treated group	13.37 ± 0.70	13.15 ± 0.71	11.94 ± 0.67	

Control group: sows inseminated with the control semen doses. Treated groups: sows inseminated with the treated semen doses.

**Table 7:** Effect of the interaction between the boars and the group of sows on litter size and the number of live-born and weaned piglets.

Boar	Sows	Litter size	Live-born	Weaned	*p*-value
1	Control group	14.52 ± 1.01	14.10 ± 1.02	11.92 ± 0.96	Group * boar > 0.05
	Treated group	13.79 ± 1.01	13.47 ± 1.03	11.36 ± 0.97	
2	Control group	14.09 ± 1.24	14.15 ± 1.26	12.66 ± 1.19	
	Treated group	11.90 ± 1.58	11.87 ± 1.60	11.22 ± 1.51	
3	Control group	14.09 ± 1.84	10.71 ± 1.86	11.10 ± 1.76	
	Treated group	12.39 ± 1.69	12.06 ± 1.71	12.30 ± 1.61	
4	Control group	14.09 ± 1.69	14.33 ± 1.71	13.29 ± 1.61	
	Treated group	15.40 ± 2.06	15.20 ± 2.09	12.89 ± 1.97	

Control group: sows inseminated with the control semen doses. Treated groups: sows inseminated with the treated semen doses.

**Table 8:** Effect of boar on litter size and the number of live-born and weaned piglets.

Boar	Litter size	*p*-value	Live-born	*p*-value	Weaned	*p*-value
1	14.15 ± 0.86	= 0.07	13.78 ± 0.87	> 0.05	11.64 ± 0.82	> 0.05
2	13.00 ± 1.17		13.01 ± 1.19		11.94 ± 1.12	
3	11.61 ± 1.39		11.38 ± 1.41		11.70 ± 1.33	
4	14.77 ± 1.48		14.77 ± 1.48		13.09 ± 1.40	

There was no effect of inseminating sows with semen doses lacking antibiotics on subsequent reproductive performance (Table 9). Table 9 shows the fertility data from the first post-experimental AI after the study’s completion.

**Table 9:** Fertility data from the first post-experimental AI after the study’s completion.

Variable	
Litter size	14.45 ± 0.37
Live-born	13.79 ± 0.39
Weaned	10.66 ± 0.32

## 4 Discussion

This is a follow-up study based on a previous *in vitro* study (6), in which boar semen prepared using a single-layer centrifugation protocol with a low-density colloid (LSLC) successfully removed or minimized bacterial contamination without the use of antibiotics. Therefore, the aim of the present study was to investigate the effectiveness of the same LSLC method for processing whole ejaculates during boar semen dose preparation under field conditions. The subsequent reproductive performance of the sows inseminated with antibiotic-free semen was also investigated. The *in vivo* fertility outcomes, such as pregnancy rate, farrowing rate, litter size, and the number of live-born and weaned piglets, were compared between the sows inseminated with LSLC boar semen doses without antibiotics and those inseminated with conventional semen doses prepared in extenders containing antibiotics.

Sperm quality was not impaired by LSLC since there were no differences between the control and treated semen doses for any of the CASA-assessed motility variables. This finding is in agreement with those of Morrell et al. (6), who found no differences between centrifuged samples with LSLC and untreated semen samples and hypothesized that these results indicate no impairment of *in vivo* fertility. It is interesting that under the experimental conditions of the present study, although higher pregnancy and farrowing rates were noticed in the sows included in the control group compared to those in the treated group, there were no differences in litter size and the number of live-born and weaned piglets between the two groups. In addition, the farrowing rate and the number of live-born piglets were different between the two rounds. For the 22 sows included in both rounds, a crossover analysis indicated that farrowing rates were the same for the sows inseminated with the control semen doses in round A and the treated semen doses in round B, or vice versa (data not shown). This is an interesting finding since it suggests that the ability of the sows to become pregnant again after insemination with semen doses without antibiotics was not impaired. As indicated, the values for litter size and the live-born piglets were lowest for boar 3, although these differences were not statistically significant. With respect to the limited number of boars (the total number of boars: 4; those with low fertility: 1) and the findings of the crossover analysis, an interaction between the boar and sow may be possible, but further investigation is needed.

The findings of the present study do not support a statistically significant effect of boar on *in vivo* fertility. However, litter size (as a tendency) and the number of live-born piglets (at a numerical level) were lowest for boar 3 compared to the other three boars. Based on the analysis of the CASA-assessed motility variables, a boar effect on motility and kinematics was also apparent. Inter- and intra-boar variability are well established (13). The variability among boars or boar ejaculates (14) is related to differences in the capacity of boar spermatozoa to withstand semen handling of different protocols or techniques, such as liquid preservation (15), cryopreservation (16), sex-sorting process (17), or IVF (18). Therefore, boars can be characterized as suitable for particular procedures, such as good or poor freezers, or suitable for IVF in terms of polyspermy. In this regard, the same approach could be applied when selecting boars for the LSLC technique. This technique could be applied in the laboratory in advance to select the suitable boars before their use in artificial insemination.

Under the experimental conditions of the current study, the treatment of boar semen with a single-layer centrifugation protocol using a low-density colloid (LSLC) could be a promising method for bacterial control in boar semen doses, as the results support acceptable *in vivo* fertility outcomes and warrant further validation. Undoubtedly, in the case of a pig farm, this technique adds an extra step to boar semen dose production, and it could be time-consuming for the staff of a pig farm, requiring training courses, investment in equipment, and the cost of the colloid itself. The preparation of AI boar semen doses at artificial insemination centers could make the application of this technique more feasible. However, cost remains an issue for AI centers, as the price of AI semen doses prepared using the LSLC technique is higher (approximately US$ 2per dose) compared to conventional methods, although this calculation did not take into account economies of scale (19). Taking into consideration the current scientific trend of finding alternatives to minimize the use of antibiotics, especially in animal breeding, the distribution of boar semen AI doses that meet this requirement could be an additional quality criterion. Undoubtedly, it is not only science but also the market that plays a role. Therefore, the extra cost could be justified, as AI semen doses produced using the LSLC technique are a state-of-the-art product that satisfies modern requirements for alternatives to antibiotics. This final argument could be a point to highlight the differences or to discuss the pros and cons of AI semen doses with or without antibiotics, especially if new rules are established in the future to encourage the restriction of the use of antibiotics in animal breeding.

## Data Availability

The raw data supporting the conclusions of this article will be made available by the authors, without undue reservation.

## References

[1] Kuster CE Althouse GC. The impact of bacteriospermia on boar sperm storage and reproductive performance. Theriogenology. (2016) 85:21–6. doi: 10.1016/J.THERIOGENOLOGY.2015.09.049, PMID: 26525397

[2] Waberski D Luther AM Grünther B Jäkel H Henning H Vogel C. Sperm function in vitro and fertility after antibiotic-free, hypothermic storage of liquid preserved boar semen. Sci Rep. (2019) 9:1–10. doi: 10.1038/s41598-019-51319-1 31611589 PMC6791940

[3] Salamon S Maxwell WMC. Storage of ram semen. Anim Reprod Sci. (2000) 62:77–111. doi: 10.1016/S0378-4320(00)00155-X, PMID: 10924821

[4] World Health Organization. Antimicrobial resistance: no action today, no cure tomorrow (2011). Available online at: https://www.who.int/director-general/speeches/detail/antimicrobial-resistance-no-action-today-no-cure-tomorrow (Accessed March 29, 2025).

[5] Morrell JM Klein C Lundeheim N Erol E Troedsson MHT. Removal of bacteria from stallion semen by colloid centrifugation. Anim Reprod Sci. (2014) 145:47–53. doi: 10.1016/J.ANIREPROSCI.2014.01.005, PMID: 24485764

[6] Morrell JM Núñez-González A Crespo-Félez I Martínez-Martínez S Martínez Alborcia MJ Fernández-Alegre E. Removal of bacteria from boar semen using a low-density colloid. Theriogenology. (2019) 126:272–8. doi: 10.1016/J.THERIOGENOLOGY.2018.12.028, PMID: 30594102

[7] Al-Kass Z Spergser J Aurich C Kuhl J Schmidt K Morrell JM. Effect of presence or absence of antibiotics and use of modified single layer centrifugation on bacteria in pony stallion semen. Reprod Domest Anim. (2019) 54:342–9. doi: 10.1111/RDA.13366, PMID: 30351456

[8] Cojkic A Hansson I Johannisson A Axner E Morrell JM. Single layer centrifugation as a method for bacterial reduction in bull semen for assisted reproduction. Vet Res Commun. (2024) 48:39–48. doi: 10.1007/S11259-023-10178-Y, PMID: 37479850 PMC10811171

[9] Lacalle E Martínez-Martínez S Fernández-Alegre E Soriano-Úbeda C Morrell JM Martínez-Pastor F. Low-density colloid centrifugation removes bacteria from boar semen doses after spiking with selected species. Res Vet Sci. (2023) 158:215–25. doi: 10.1016/J.RVSC.2023.03.024, PMID: 37031470

[10] Lacalle E Fernández-Alegre E Soriano-Úbeda C Martínez-Martínez S Domínguez JC González-Montaña JR. Single layer centrifugation (SLC) for bacterial removal with Porcicoll positively modifies chromatin structure in boar spermatozoa. Theriogenology. (2023) 201:95–105. doi: 10.1016/J.THERIOGENOLOGY.2023.02.017, PMID: 36857978

[11] Morrell JM van Wienen M Wallgren M. Single layer centrifugation can be scaled-up further to process up to 150 mL semen. ISRN Vet Sci. (2011) 2011:1–6. doi: 10.5402/2011/183412, PMID: 23738111 PMC3658788

[12] Watson PF Behan JR. Intrauterine insemination of sows with reduced sperm numbers: results of a commercially based field trial. Theriogenology. (2002) 57:1683–93. doi: 10.1016/S0093-691X(02)00648-9, PMID: 12035978

[13] Gil MA Almiñana C Roca J Vázquez JM Martínez EA. Boar semen variability and its effects on IVF efficiency. Theriogenology. (2008) 70:1260–8. doi: 10.1016/J.THERIOGENOLOGY.2008.06.004, PMID: 18676010

[14] Parrilla I del Olmo D Sijses L Martinez-Alborcia MJ Cuello C Vazquez JM. Differences in the ability of spermatozoa from individual boar ejaculates to withstand different semen-processing techniques. Anim Reprod Sci. (2012) 132:66–73. doi: 10.1016/J.ANIREPROSCI.2012.04.003, PMID: 22554791

[15] Kommisrud E Paulenz H Sehested E Grevle IS. Influence of boar and semen parameters on motility and acrosome integrity in liquid boar semen stored for five days. Acta Vet Scand. (2002) 43:49–55. doi: 10.1186/1751-0147-43-49 12071116 PMC1764181

[16] Roca J Hernández M Carvajal G Vázquez JM Martínez EA. Factors influencing boar sperm cryosurvival1. J Anim Sci. (2006) 84:2692–9. doi: 10.2527/JAS.2006-094 16971570

[17] Alkmin DV Parrilla I Tarantini T Parlapan L del Olmo D Vazquez JM. Intra- and interboar variability in flow cytometric sperm sex sorting. Theriogenology. (2014) 82:501–8. doi: 10.1016/J.THERIOGENOLOGY.2014.05.008, PMID: 24930604

[18] Suzuki H Saito Y Kagawa N Yang X. In vitro fertilization and polyspermy in the pig: factors affecting fertilization rates and cytoskeletal reorganization of the oocyte. Microsc Res Tech. (2003) 61:327–34. doi: 10.1002/JEMT.10345, PMID: 12811737

[19] Morrell JM Cojkic A Malaluang P Ntallaris T Lindahl J Hansson I. Antibiotics in semen extenders – a multiplicity of paradoxes. Reprod Fertil Dev. (2024) 36:218. doi: 10.1071/RD23218, PMID: 38447204

